# Category Structure and Categorical Perception Jointly Explained by Similarity-Based Information Theory

**DOI:** 10.3390/e20070527

**Published:** 2018-07-14

**Authors:** Romain Brasselet, Angelo Arleo

**Affiliations:** 1 Cognitive Neuroscience Sector, SISSA, Via Bonomea 265, 34136 Trieste, Italy; 2 Center for Brain and Cognition, Computational Neuroscience Group, Department of Information and Communication Technologies, Universitat Pompeu Fabra, 08018 Barcelona, Spain; 3 Sorbonne Université, INSERM, CNRS, Institut de la Vision, 17 rue Moreau, F-75012 Paris, France

**Keywords:** goodness, categorical perception, perceptual magnet, information theory, perceived similarity

## Abstract

Categorization is a fundamental information processing phenomenon in the brain. It is critical for animals to compress an abundance of stimulations into groups to react quickly and efficiently. In addition to labels, categories possess an internal structure: the goodness measures how well any element belongs to a category. Interestingly, this categorization leads to an altered perception referred to as categorical perception: for a given physical distance, items within a category are perceived closer than items in two different categories. A subtler effect is the perceptual magnet: discriminability is reduced close to the prototypes of a category and increased near its boundaries. Here, starting from predefined abstract categories, we naturally derive the internal structure of categories and the phenomenon of categorical perception, using an information theoretical framework that involves both probabilities and pairwise similarities between items. Essentially, we suggest that pairwise similarities between items are to be tuned to render some predefined categories as well as possible. However, constraints on these pairwise similarities only produce an approximate matching, which explains concurrently the notion of goodness and the warping of perception. Overall, we demonstrate that similarity-based information theory may offer a global and unified principled understanding of categorization and categorical perception simultaneously.

## 1. Introduction

Categorization is a cognitive process through which a large number of *items* (objects, events, stimuli; sometimes referred to as *instances* or *exemplars*) are grouped into a few classes. It is a bottleneck from an immensely complex world to relevant representations and actions [1] and thus it allows us to react quickly and communicate efficiently. Categorizing amounts to compressing the perceived world by putting the same label on many items, thereby preserving the relevant information and discarding the irrelevant one. Importantly, such a binary perception has been shown to be suboptimal [2], since categories to which the item may belong with weaker probabilities are discarded. From a relationist viewpoint, categorization consists in considering as *similar* two items in a category and as *different* two items in different categories. According to this view, Rosch [3] gives the following definition “To categorize a stimulus means to consider it (…) not only equivalent to other stimuli in the same category but also different from stimuli not in that category”.

In addition, categories also have an internal structure: each item has its own measure of how well it represents its category, which is called *goodness* [4,5,6] (also referred to as *membership* or *typicality*). The item with the largest goodness in a category is called the prototype of this category [5]. This internal structure plays an important role in the speed of classification [4], exemplar production [7], or two-item discrimination [8]. The ontology of this graded internal structure is dependent on both the frequency of instantiation of an item as a member of the category [7,9,10] and the pairwise similarity structure [6,8]. The prototype has in general a large frequency and it is similar to other items of the category [5]. For instance, although it is a frequently mentioned bird, a chicken is not judged as being very similar to other birds and thus it has low goodness [11].

Interestingly, categorization is not only a bottom-up process as it bears effects on perception. One of these effects is called *categorical perception* [12]: items within a category are harder to discriminate than items in different categories, even if they are separated by the same physical distance (physical distance here means distance in the relevant physical space: frequency, amplitude, wavelength… or any metric space in which the items may be embedded). In other words, within-category discrimination is reduced while between-category discrimination is enhanced. Discrimination performance is only slightly better than category identification, though the within-category subtleties can be observed through the reaction times [13]. This effect has been observed on similarity between faces [14,15], colors [16,17], or speech sounds [12].

An additional effect is that prototypes of a category pull other items in the category toward themselves, which is called the *perceptual magnet* effect [18]. Items at the center of a category are perceived closer than at the border of a category. Iverson and Kuhl have shown the warping of the perceptual space using multi-dimensional scaling [19,20]. This effect is known to be asymmetrical [8,18,19]: in a two-stimuli discrimination task, if a prototype is presented before a non-prototype, the discrimination results are poorer than when the non-prototype is presented first. For a thorough review of these phenomena in natural and artificial categories, as well as an account of them through Bayesian inference, alternative to our explanation, we refer the reader to Feldman et al. [21].

The interactions between category boundaries, category structures, categorical perception, and perceptual magnet are still debated. It is so far unknown whether one of them is more fundamental and entails the others as consequences. We take here a holistic approach and attempt to show that they are all facets of categorization. All categories indeed have an internal structure [11], the notion of goodness thus appears inseparable from categorization. In addition, categorical perception has been observed commonly, although with some variability [22].

A large body of work already attempted at modeling categorical effects. Of particular interest to us here is the context theory of classification [23,24] that takes into account the similarities between items and proposes a measure comparing within-category similarities to between-category similarities. Our work will naturally lead us to consider a very similar measure, which we will interpret in information-theoretic terms. We will also consider the work by Bonnasse-Gahot and Nadal [25] that is the closest to ours in terms of explanations of categorical phenomena. They give an information-theoretic account of categorical perception and perceptual magnet as optimization of neural coding of categories.

Here, we aim at explaining altogether the structure of categories and the categorical perception phenomena by applying a recently introduced optimization principle for information processing. We start with well-defined categories whose items appear with uniform or bell-shaped frequencies. Following Tversky and Gati [8], we model human discrimination between two items with a notion of perceived similarity that can take any values in the range [0,1]: two items with a similarity of 1 are perceived as identical while two items with a similarity of 0 are perceived as different. Therefore, this work makes use of frequencies and pairwise similarities as fundamental features of our cognitive processes. We then use a new information-theoretic principle for optimizing the pairwise similarity values.

As information theory is a suitable tool and a very efficient framework to understand information processing in the brain [26,27,28] (and references therein), we account for categorical perception by applying information theory to categorization. We use a recently introduced version of information theory integrating pairwise similarities [29], whose formulation naturally merges reliability of information transmission and compression of the stimulus space. In this sense, the present work can be compared to that of Bonnasse-Gahot and Nadal [25] who used information theory to find optimal neural population codes to encode categories and account for some aspects of categorical perception.

In a general way, the process of learning categories can be described with two opposing strategies, as stated by Pothos [30] in the case of Artificial Grammar Learning, “the similarity/rules/association and the *information premise*”, arguing that a shortcoming of information theory to understand cognition is its lack of tools for understanding representations. We hope to demonstrate the possibility to develop such tools. In this paper, we provide a theory that naturally encompasses the two approaches, thereby attempting to show that they may not be as much in opposition as they seem. We build on a recent modification of information theory that involves quantities for representations, namely, pairwise similarities between items considered.

Armed with this principle, we derive the internal structure of categories and categorical perception simultaneously. Indeed, the formulation naturally involves, for each element, an average of its similarities weighted by the probabilities of every other element within the category, which is readily interpreted as how similar one object is to the others on average, and it is shown to match the notion of goodness [5].

One could understand our method the following way: suppose a subject is trying to perfectly categorize an ordered set of stimuli. By perfectly, we mean that all specificities of the stimuli beyond the category are forgotten. This amounts exactly to maximizing the information while minimizing the equivocation. Now let us in addition suppose that there are limits to the ability of a subject to achieve this perfect categorization because of finite discrimination capacity. We choose to model the latter by similarity functions. The framework of similarity-based information theory (SBIT) is a very natural one to use here as it integrates similarities to information theory (IT).

## 2. Methods

### 2.1. Item Space and Categories

Items S={s} are considered on a one-dimensional axis and grouped into a set of categories C={c}. These categories represent the pre-existing ideal categorization that the similarity measures have to emulate. These do not only depend on the observer: as in the case of colors, they can be very influenced by culture [31] (we come back to this issue in the discussion). As categorical perception effects are ubiquitous and appear in many modalities, we make no specific assumptions about the distribution of items inside a category. For example, it appears highly reasonable to consider light wavelengths to be uniformly distributed [32]. On the contrary, it is known that, in a given language, speech sounds are well defined but are modulated by noise or idiosyncrasies. This results in an ensemble of bell-shaped categories. Thus, for the sake of completeness, we will consider two extreme cases: one with contiguous uniformly distributed categories and another with bell-shaped categories. We also consider the case of bimodal categories. These three cases represent extreme cases in between which other one-dimensional cases will exist. In all cases, we will refer to their width (or, equivalently, the number of items) as *W*. We thus believe we exhibit exhaustively the phenomena of interest on all potential distributions of categories. The extension to higher-dimensional cases is straightforward and leads to qualitatively similar behaviors.

### 2.2. Similarity Functions

We define a similarity function $\phi_{s}\left(s^{\prime }\right)$, between the item $s^{\prime }$ and the reference item *s*, that takes values in the interval [0,1] and describes how similar the item $s^{\prime }$ is to reference item *s*. We use a biologically reasonable constraint on the similarity function: at each point, it has to be a non-strictly decreasing function of the physical distance (e.g., Heaviside, bell-shaped, Gaussian, triangular). This is a very light constraint and seems, to the best knowledge of the authors, the only sensible behavior a similarity function can adopt. Unless we twist words and concepts heavily, two very different objects cannot be construed as more similar than two less different objects.

We choose a triangular pairwise similarity function given by $\phi_{s}\left(s^{\prime }\right)=1-\frac{|s-s^{\prime }|}{\sigma (s)}$ when $|s-s^{\prime }|<\sigma \left(s\right)$ and 0 otherwise. The width function σ(s) takes a different value at each point in the item space and is the only free parameter to be tuned. The variation of the similarity width is akin (although not necessarily fully equivalent) to the attention-specific warping of the stimulus space in the context theory of classification or to the variation of the widths of the neural tuning curves in Bonnasse-Gahot and Nadal [25]. Note the potential asymmetry here—if $\sigma \left(s\right)\neq \sigma \left(s^{\prime }\right)$, then $\phi_{s}\left(s^{\prime }\right)\neq \phi_{s^{\prime }}\left(s\right)$. The choice of a triangular function over another type of bell-shaped function is motivated by the empirical fact that the results are not qualitatively affected by the choice of similarity function, as long as it is not singular in any way (a condition akin to the one in [25] about the smoothness of the tuning curves), and by the ease of the mathematical treatment of the triangular function, which allow us to directly compare simulations and calculations.

Now that we are equipped with such similarity functions, we need to define an optimization principle. To do so, we make use of the similarity-based mutual information between the set of categories and the items with their pairwise similarities.

### 2.3. Similarity-Based Information Theory

Here, we recall the main concepts of similarity-based information theory (SBIT), a well-established theory with a versatile, albeit recent, history.

SBIT can be seen as an extension of IT, which is a framework to quantify statistical dependencies between variables, mainly through the definition of the entropy of a distribution, that quantifies its uncertainty. While IT takes into account only the probabilities of events or items, it discards entirely all other features of the dataset, in particular to what extent two items are similar or not. SBIT is precisely an attempt to extend IT by incorporating similarities in the very definition of entropy.

A similarity-based entropy was first introduced as a measure of biodiversity. In this field, the original concept was Rao’s quadratic entropy that incorporates the distance between two species [33]. Ricotta and Szeidl [34] proposed a family of similarity-based Tsallis entropy, while Leinster and Cobbold [35] introduced a family of similarity-based Renyi entropy. Both Tsallis and Renyi entropies entail Rao’s quadratic entropy as a special case. The similarity-based Renyi entropy reduces to a similarity-based Shannon entropy for particular values of its free parameter. The rationale for these concepts is that entropies only deal with probabilities but do not take into account potential similarities between items. Therefore, in the field of biodiversity, a population of canopy butterflies or a mixed population of canopy and understorey butterflies with the same probability distribution would have the same Shannon entropy, but not the same similarity-based entropy. Accounting for the similarities between species sheds a new light on the meaning of biodiversity.

Among all these entropies, the advantage of Shannon entropy is that there exists an unequivocal definition of conditional entropy [36,37], thereby allowing the mutual information between two variables to be defined. This mutual information is readily interpreted as a reduction in uncertainty about a variable when the other is known. This similarity-based mutual information was introduced and applied to neural coding [29], and we use it here. We wish to emphasize how the theory used here is grounded in other fields of study and applied without ad hoc extensions to the topic of cognition.

Our hypothesis is that information processing has to focus on thoroughly discriminating some pairs of items, thereby guaranteeing information transmission, but, simulteaneously, it also has to overlook differences between other pairs, i.e., compress the stimulus set, which is assessed by the conditional entropy. This is what is referred to as categorization. This of course builds heavily on previous work and is not a new way of looking at categorization. Neither is the use of information theory to do so (see for example [25,38]). However, we address it in a new manner, using an extension of information theory that allows us to account for a set of phenomena that, to our knowledge, was never explained by a single model.

We first define (following Brasselet et al. [29]) the specific similarity-based entropy h(s) (also known as *surprise*) :

(1) $$\begin{array}{ccc} h(s) & = & -\log g(s), \end{array}$$

where $g\left(s\right)=\sum_{s^{\prime }}p\left(s^{\prime }\right)\phi_{s}\left(s^{\prime }\right)$, which was defined similarly by Ricotta and Szeidl [34], Leinster and Cobbold [35]. Note here that this quantity g(s) is a sum of probabilities weighted by similarities with *s* and is therefore always comprised between 0 and 1. It can therefore be thought of as “the probability of item *s* or another item $s^{\prime }$ similar to it”. A high value of h(s) means that it is surprising to observe the item *s* or another item $s^{\prime }$ similar to it. On the contrary, a small value tells us it is not surprising to observe it. This may happen when:

- the probability of *s* is itself large,
- another item $s^{\prime }$ with large probability is very similar to *s*,
- many low-probability items have large similarity with *s*.

In classical IT, only the first case exists.

Note that the corresponding entropy can be obtained by averaging the specific entropies over all items, $H\left(S\right)=\sum_{s}p\left(s\right)h\left(s\right)$.

Mathematically, the behavior of the similarity-based entropy is well-understood [29,35]. In the extreme case where the similarities are 0 everywhere, except for $s=s^{\prime }$, we recover the probability of *s* and thus the original definition of Shannon surprise and Shannon entropy. Similarity-based information theory thus departs from Shannon theory that considers all items *s* to be different with no gradation. In such cases, the arguments of the logarithm cannot be taken as probabilities, since they do not sum to 1. However, the behavior of h(s) is smooth as a function of the similarity matrix as it departs from the identity matrix, and eventually reaches h(s)=0 when all the similarities are equal to 1, akin to the classical Shannon case where all items are indistinguishable (in the case of binning continuous variables for example). The similarity-based entropy thus takes values in the same range as Shannon entropy and has a natural interpretation. More properties are given in the references previously mentioned in this paragraph and we follow them in calling this quantity “entropy” as it meets all the criteria established by [39]. Researchers in ecology defined it to account for potential genetic similarities between species, while, in neuroscience, it was purposefully defined to account for similarities between percepts or representations.

Once we made the first step towards extending entropy with similarities, the definition of all the other quantities naturally follows. We can then define the specific conditional entropy h(s|C) [40]:

(2) $$\begin{array}{ccc} h(s|C) & = & -\sum_{c}p\left(c|s\right)\log g\left(s|c\right), \end{array}$$

where $g\left(s|c\right)=\sum_{s^{\prime }}p\left(s^{\prime }|c\right)\phi_{s}\left(s^{\prime }\right)$. This quantity can be thought of as “knowing the value of variable *c*, the probability of item *s* or another item $s^{\prime }$ similar to it”. This specific conditional entropy can be thought as the uncertainty of items or items similar to them within a category *c*.

Note that, again, the corresponding conditional entropy can be obtained by averaging the specific entropies over all items $H\left(S|C\right)=\sum_{s}p\left(s\right)h\left(s|C\right)$. The conditional entropy is also known as *equivocation* because it measures how items are confused or, in other words, how the mapping from *s* to *c* is equivocal.

As usual, the specific similarity-based mutual information i(s;C) is defined as the difference between the specific entropy and conditional entropy i(s;C)=h(s)−h(s|C) and reads:

(3) $$i\left(s;C\right)=\sum_{c}p\left(c|s\right)\log\frac{g(s|c)}{g(s)}.$$

This information increases with the argument of the logarithm, which is positive when an item is more probable within the category or more similar to other items within the category (g(s|c)) than it is probable overall or similar to other items items overall (g(s)). Therefore, for a given item with fixed probability, the information is large when the item is similar to items within the category but not with items outside. As usual, we recover the information by summing the specific information over the items: $I\left(S;C\right)=\sum_{s}p\left(s\right)i\left(s;C\right)$.

Importantly, all of these quantities reduce to Shannon specific entropies and specific mutual information in the case where the similarity function $\phi_{s}\left(s^{\prime }\right)$ is equal to 0 except for identity $s=s^{\prime }$.

### 2.4. Optimization Principle

An important feature of the previously defined entropy h(S), conditional entropy h(s|C) and mutual information i(s;C) is that they all depend on the similarity function $\phi_{s}\left(s^{\prime }\right)$. Large values of similarities, i.e., items are very much alike, will lead to low values of h(s), h(s|C) and i(s;C). Conversely, low values of the similarities, i.e., all items appear as different, will increase the values of h(s), h(s|C) and i(s;C). In our case, following Rosch’s suggestion, we want to guarantee high similarities between objects from the same categories and low similarities between objects from different categories. High similarities inside categories amount to minimizing the conditional entropy while low similarities between categories amount to maximizing information between categories and items.

As is usually done in models of categorization, we implement the trade-off between maximizing information and minimizing conditional entropy by introducing an objective function involving a free trade-off parameter α:

(4) $$q\left(s;C\right)=i\left(s;C\right)-\alpha \frac{h(s|C)}{h_{0}}.$$

(In the specific case of Brasselet et al. [29], α was chosen to be infinity). Note that this trade-off parameter is akin to the one we find in rate distortion theory (RDT) or in the information bottleneck (IB). The addition of our model is the integration of similarities. Just like in RDT, the objective is to minimize a cost function subject to an upper bound on the information, just like in IB, the objective is to maximize the information between two sets while minimizing that between one of these sets and an encoder, in SBIT, the objective is to maximize the information while minimizing the equivocation. To go further, we can compare this objective function with the one used by Sims et al. [38]:

(5) $$\min_{p(y|x)}E\left[f\left(y-x\right)+P\left(C_{x}\neq C_{y}\right)\right],I\left(x,y\right)<C.$$

Maximizing the information between the item and its category is akin to not mistaking an item for another one. Minimizing the equivocation is akin to bounding the information.

We believe that a trade-off parameter is a necessary feature of any model of categorical perception. Indeed, there is a need for a compromise between compression (categorization) and information conveyance that may depend on the subject or the task at hand. This can only be captured by a quantity akin to a trade-off parameter.

A technical note about the quantity $h_{0}$ is in order here. In the present paper, we discuss the discrete case, but we aim at providing a framework that accomodates both discrete and continuous cases. Unlike information, conditional entropy is not independent from discretization. Therefore, we have to regularize it by a measure that depends commensurately on the discretization. This also allows the specific values of α to be independent from discretization. For a given problem, however, only the ratio between α and $h_{0}$ matters, so α can be redefined in units of $h_{0}$. In the sequel, we choose $h_{0}=\log\left(W\right)$. Note that we are only concerned with positive values of α since we are looking at information maximization and conditional entropy minimization.

We apply this method to a categorization protocol and we use both a mathematical and computational approach. The problem is in general solvable analytically and we provide a solution in the case of uniform categories and triangular similarities in Appendix A. In the main body of the paper, we treat the problem computationally. As the maximization of the objective function q(s;C) can be done independently at each point of the item space, we optimize the similarity measure by making an exhaustive search in the width space σ(s) and by selecting the optimal value for the objective function.

## 3. Results

### 3.1. Non-Overlapping Uniform Categories

We first consider items S={s} characterized by a single parameter that is distributed uniformly over a single axis. Items *S* are grouped into *N* categories C={c} of width *W* (see Methods). For the sake of ease of explanation and interpretation, all the categories have the same size, though it is not a necessary condition. We optimize the objective function that maximizes information and minimizes the conditional entropy (see Methods). At each point, we compute the value of the similarity-based entropy, conditional entropy, information as well as objective function for each value of the similarity width σ(s). We select the value of σ(s) that maximizes the objective function. The particular results shown here are for n=10 categories of width W=100, for a total of 1000 items.

**Figure 1:**
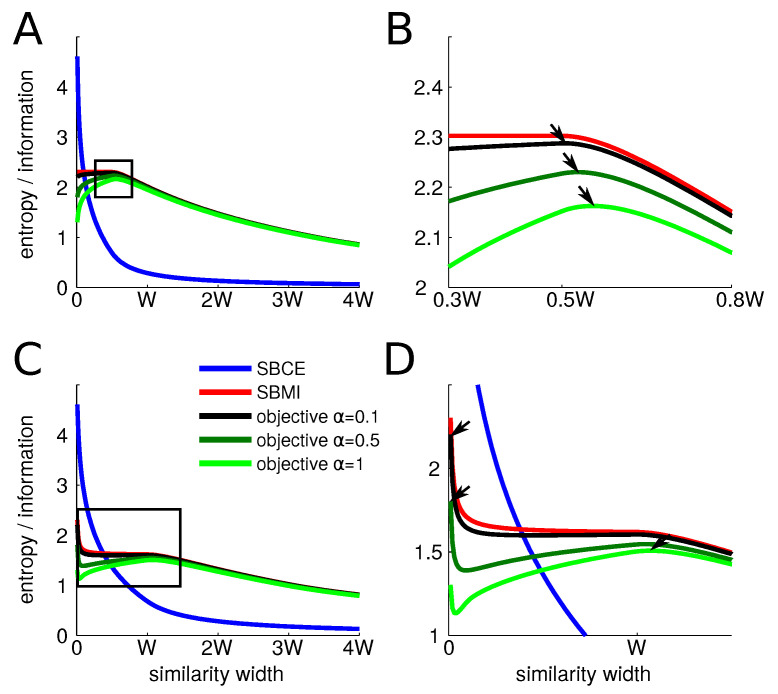
The mutual information SBMI, conditional entropy SBCE and objective function at the center (**A**,**B**) and border (**C**,**D**) of a category of width *W* depending on the similarity width with triangular similarity measures. Since information and entropies are always positive, larger values of α always lead to lower objective functions. The right panels (**B**,**D**) are magnifications of the rectangles depicted in the left panels (**A**,**C**). Arrows indicate the maxima of the objective functions. We observe that the maximum of the objective function at the center is always larger than W/2 and increases slowly with trade-off parameter α, while the maximum at the border is very small for small values of α and then undergoes an abrupt increase. These results are obtained for categories of width *W*.

We provide the behavior of the different entropies at the center and at the boundary of categories as we explore the possible values of the similarity width. As the width of the similarity function increases, both the information and the conditional entropy decrease, although they do so at different paces. At the center of a category (Figure 1A,B), on the one hand, the conditional entropy starts at log(W), it undergoes a sharp drop as the width increases from 0 to W/2 and then decreases more slowly. On the other hand, for values of the width smaller than W/2, the information remains at its maximum of log(N) as all similarities between items from different categories remain zero. It only starts decreasing for values larger than W/2. Thus, the objective function always finds its maximum at a value larger than W/2. As the trade-off parameter α increases, the optimal width also does.

However, on the border of a category (Figure 1C,D), the behavior is radically different. The conditional entropy starts at log(W) and undergoes a drop as well as the width increases but less sharp than at the center as the similarity is increased with fewer items of the same category at a given similarity width. The information starts also at log(N) when the width is very small as there is no positive similarity between items of different category. However, as soon as the width increases, similarities between items of different categories increase and the information consequently drops and plateaus to log(N/2). This value comes from the fact that the similarity function essentially mixes two categories. Then, when the similarity width overpasses *W*, confusion arises with even more categories and the information decreases steadily even more. Thus, for low values of α, the optimal width is zero. As α increases, there is a sudden transition of the optimal width from 0 to *W* that then steadily keeps on increasing. More details and computations are given in Appendix A. We give only a summary of the final results here. We find that the optimal value of the width σ at the center of a category, i.e., at (m+1/2)W, is:

(6) $$\sigma_{opt}=\frac{W}{2}\left(1+\frac{\alpha }{2\log W}\right).$$

This value is always larger than (or equal to) W/2. The intuitive reason for this is that, up to W/2, it only increases similarity with items within the category, and thus does not reduce information while decreasing equivocation. In addition, note that, to a good approximation, $\sigma_{opt}$ is proportional to *W*, meaning that the similarity function scales with the category. Note that this value also grows linearly with α, for *W* fixed. This is because the more we focus on minimizing the equivocation, the larger the width has to be.

We also find that the similarity width at the boundary of a category (i.e., the item closest to a boundary) is:

(7) $$\begin{array}{ccc} \sigma_{opt}= & 1, & \mathrm{if}\;\alpha <\alpha_{th}, \end{array}$$

(8) $$\begin{array}{ccc} \sigma_{opt}= & \left(W-1\right)\left(1+\frac{\alpha }{2\log W}\right), & \;\mathrm{otherwise}. \end{array}$$

When α is small, i.e., when little focus is on minimizing the equivocation, the optimal width is 1. Indeed, any departure from this would create confusion between the element *s* of category *c* and a neighboring category $c^{\prime }$. When α is large, the optimal width is always larger than W−1. This is due to the possible reduction in equivocation yielded by extending the similarity function to encompass all the elements in category *c* and, collaterally, those of a neighbouring category $c^{\prime }$.

Before turning to more complete computational simulations, we see already that the perceptual magnet effect will happen only for low values of α, when the optimal width at the center is larger than that at the boundary of a category.

**Figure 2:**
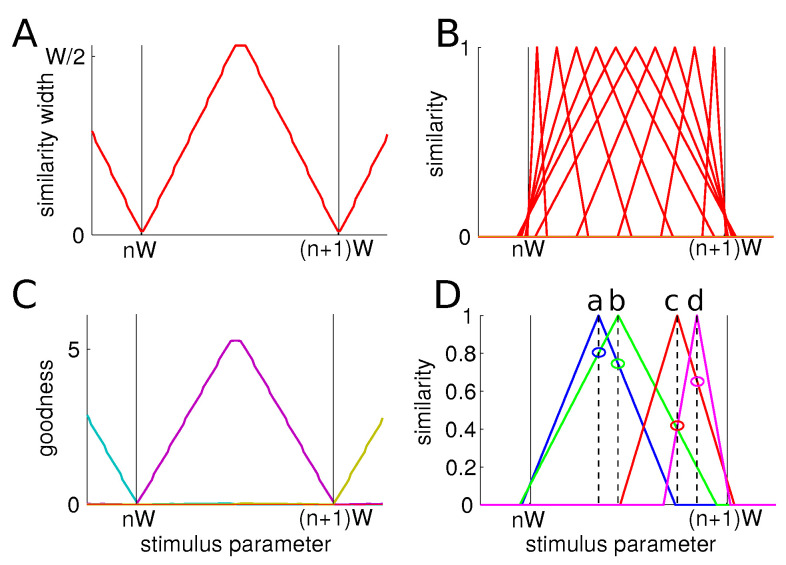
Results for categories of width *W* and trade-off parameter α=0.5. (**A**) similarity width for all members of a category and (**B**) similarity functions of selected members. The further away from the the center of the category, the smaller the similarity width and the narrower the similarity function; (**C**) goodness of all members of a category. Members at the center have larger goodnesses. In the case of uniform categories, they mimick the similarity widths; (**D**) similarities between two equidistant pairs of members either at the center or a the border of a category. We see that a pair of items (a and b) near the center has larger similarities than a pair (c and d) closer to the border.

We then assess the value of the similarity width across the stimulus space for a low value of the trade-off parameter, i.e., α=0.5 (Figure 2A). We recall that a low value of α means that the first objective is to maximize information and then, as a secondary objective, to minimize the conditional entropy. We observe that, in agreement with the previous results, the similarity measure width is much larger at the center of categories than at their boundaries. Examples of the similarity functions at selected places in the stimulus space are given in Figure 2B.

We also evaluate the functions g(s|c) (see Equation (2)) for all members across the stimulus space for α=0.5 (Figure 2C). They exhibit a graded behavior: central members have high values while border members have low ones. This is due to the fact that central elements have large similarity widths and thus they are considered similar to other elements of the category. In the particular case of one-dimensional uniform categories, the g(s|c) and the similarity width have the exact same behavior, but this will not be the case for other distributions, as we will see in following. The term $g\left(s|c\right)=\sum_{s^{\prime }}p\left(s^{\prime }|c\right)\phi_{s}\left(s^{\prime }\right)$ is readily interpreted as a measure of the goodness of the member *s* in category *c*.

### 3.2. Preliminary Discussion

These results collectively match cognitive effects presented earlier:

- Categorical perception: two items at a given distance are perceived as more similar if they are within a category than if they are from two different categories. Indeed, if two items a and b from the same category are presented simultaneously, their similarities are 0.75 and 0.8, while two items c and d, which are at the same physical distance, have lower similarities 0.4 and 0.65 (Figure 2D).
- Perceptual magnet: the similarity measure is wider at the center of category, so items around it are perceived as more similar to each other than two other items at other locations within the category. Finally, we observe the asymmetry effect: starting from the prototype, an object on the border appears more similar than the other way around, or $\phi_{s_{0}}\left(s_{1}\right)>\phi_{s_{1}}\left(s_{0}\right)$.

However, the perceptual magnet effect depends on the value of the trade-off parameter α. For α<0.79 (see Appendix A), the perceptual magnet effect appears, but, for large values, the similarity measure peaks in-between categories, as shown in Figure 1. When the value of α is low, the objective is mainly to maximize information. Therefore, anything that reduces information is prohibited. More precisely, having a non-nil similarity between two items of two different categories reduces information. Thus, the maximal width of a similarity is the one that keeps the similarity low between items from two different categories. However, central items of a category are more remote to other categories than off-central items. Their similarity measure can therefore safely be wider.

As α increases, minimizing conditional entropy becomes more and more important, forcing the similarity measures to be wider. At α=0.79, there is a jump in the optimal width at the border of a category: it goes from $x_{opt}=1$ to $x_{opt}>W$ (see Appendix A). At this point, the similarity width becomes larger at the border than at the center and the perceptual magnet effect disappears. These opposite behaviors at low α and large α were found in all simulations no matter the shapes of category distribution. Because our main interest is in the ability of the model to produce a categorical perception effect, in the subsequent parts, we always choose a value of α below the critical value that sees a sudden jump in the similarity width at the boundaries of categories.

### 3.3. Gaussian Categories

We performed the same analysis on a set of items distributed on a one-dimensional space. These items are organized in Gaussian categories separated by a distance W=100 and variance ν:

(9) $$p\left(s|c_{i}\right)∼N\left(W\left(i+\frac{1}{2}\right),\nu \right).$$

Again, we consider a triangular kernel whose width has to be optimized according to the similarity-based objective function. We carried the analysis with 10 values of α ranging from $5\times 10^{-10}$ to $5\times 10^{-1}$ equally spaced logarithmically.

**Figure 3:**
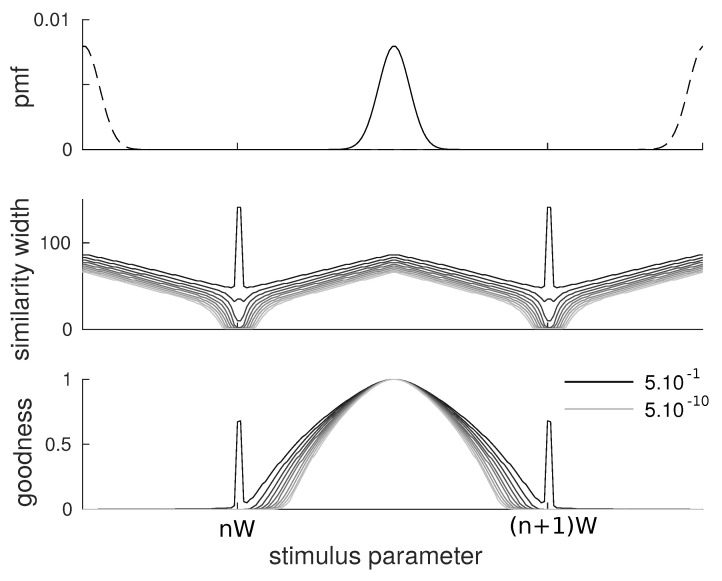
Results for Gaussian categories of variance ν=5 separated by a distance W=100 and trade-off parameter α ranging from $5\times 10^{-10}$ to $5\times 10^{-1}$. (**top**) probability mass function of the categories; (**middle**) similarity width for all members of a category. As in the uniform category case, the similarity is wider at the center than at the border, although the exact shape is more complicated; (**bottom**) goodness of all members of a category. The goodness results here from an interplay between frequencies and similarities, but it displays the expected behavior of larger goodnesses at the center.

The results are qualitatively similar to those obtained with flat categories. We observe that the similarity width (Figure 3, middle) is larger at the center of categories than at the border, although in this case, the width does not reach the minimum value as opposed to the flat category case. As for g(s|c), we also observe a qualitatively similar behavior with items at the center having larger values than items on the border (Figure 3, bottom). The shape of the goodness curve g(s|c) differs from the one of the similarity function since it also involves the Gaussian shape of categories. In particular, we observe that it drops faster when moving away from the prototype as the effects of the probabilities and distances multiply.

### 3.4. Displacement Measure

In order to quantify the warping of the perceptual space, in the spirit of Feldman et al. [21], we use a measure of the displacement of each item within its category. We define the displacement of item $s_{0}$ as the average of the positions of the other items $s_{i}$ within the category weighted by the similarity between $s_{0}$ and $s_{i}$. It reads:

(10) $$D\left(s_{0}\right)=\sum_{i}\phi_{s_{i}}\left(s_{0}\right)s_{i}.$$

**Figure 4:**
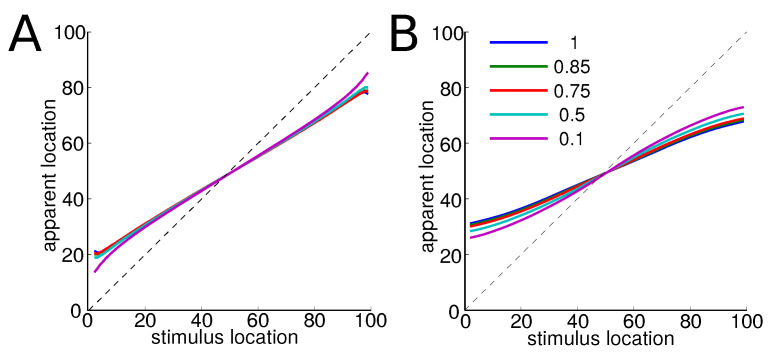
Measure of displacement of items within a category for different values of the trade-off parameter α. (**A**) uniform categories; (**B**) Gaussian categories. We observe that the more central element, the prototype, is not displaced while other items are all more displaced towards the center of the category, as they are far from the prototype. This result holds for any value of the trade-off parameter.

This is indeed a measure of where the average similar item is in the stimulus space. Therefore, this measure provides a good estimate of the position of the item in the perceptual space. For any value of the trade-off parameter α, we find qualitatively similar results, see Figure 4: the more the items are far from the center of a category, the more they are displaced towards its center. Items near the center are only slightly displaced.

The larger effect for Gaussian categories may account for the stronger perceptual magnet in discrete categories such as consonants compared to continuous, such as vowels.

### 3.5. Bimodal Categories

To assess the effect of potential bimodality of distributions, the same analysis was made on bimodal categories. The items in each category are now distributed as the sum of two Gaussians:

(11) $$p\left(s|c_{i}\right)∼N\left(W\left(i+\frac{1}{2}\right)-\delta ,\nu \right)+N\left(W\left(i+\frac{1}{2}\right)+\delta ,\nu \right).$$

**Figure 5:**
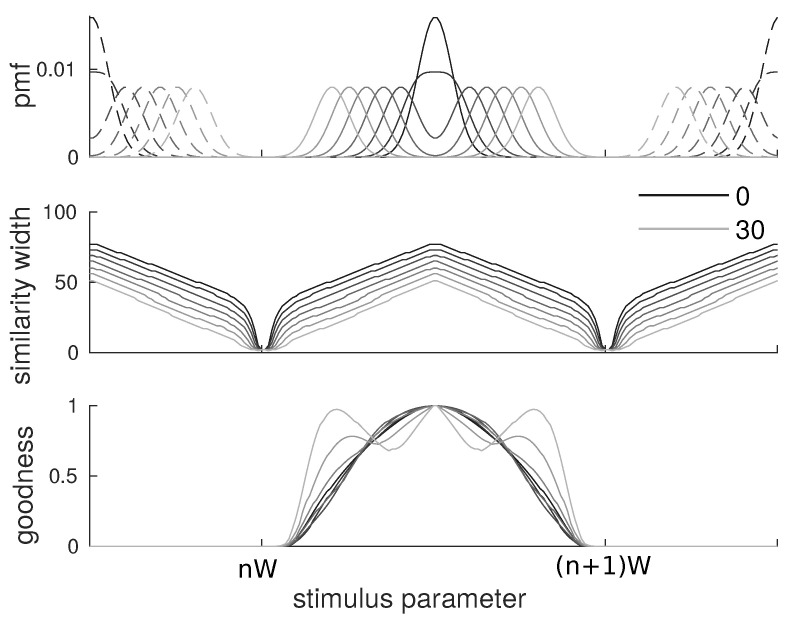
Results for bimodal categories made of two Gaussians of variance ν=5 separated by a distance δ ranging from 0 to 30. We used α=0.0005. (**top**) probability mass functions of the categories in the 7 cases; (**middle**) similarity width for all members of a category. As in the uniform and Gaussian category cases, the similarity is wider at the center than at the border; (**bottom**) goodness of all members of a category. The goodness has a more complicated behavior than in previous cases—from bell-shaped in the δ=0 case (i.e., Gaussian monomodal) to multimodal for large δ.

We kept the spacing of W=100 between categories from the previous cases and chose a variance of ν=5 for the Gaussians to have clear bimodality. To study these cases, we chose a value of $\alpha =5\times 10^{-4}$ for which the perceptual magnet effect is very clear. We display results for δ from 0 to 30 (point at which the two peaks are further apart than they are to other categories) in increments of 5: namely, the category shapes, the similarity width and the goodnesses associated. See Figure 5.

We find that, overall, the similarity width keeps the same shape as in the monomodal Gaussian case, although its absolute value decreases. For a marked bimodal case, this is due to the the absence of a central mass in the distribution, compared to the bell-shaped case. In such a case, points close to one peak of the category do not decrease equivocation much by increasing their similarity width. The similarity width would have to be very large, but then the proximity of the other category prevents them from decreasing equivocation without decreasing information.

Note that, at some point, the similarity width becomes small and thus the goodness is mostly determined by the frequency. In a strong bimodal case, it naturally makes the goodness bimodal. We are not aware of an empirical case where this is observed. This leads us to venture that such unnatural cases where categories are bimodal with their modes further apart than they are from other categories may be unmanageable. However, it gives a prediction for artificial category learning in a laboratory setting.

## 4. Discussion

First, the argument g(s|c) that appears in the logarithm of the conditional entropy behaves naturally like the goodness of any given item *s* in the category *c*. The exemplar at the center of a category has the largest g(s|c) and then qualifies as a prototype. Note that, in the uniform category case, the high goodness is only due to the internal similarity structure and not to frequencies. This matches the notion of prototype as having more attributes in common with other members of the category and less with members of other categories [5]. A posteriori, it appears natural to define the goodness of an exemplar as the average of its similarity with other exemplars: a good prototype should have features in common with most other items.

Second, among the theoretical advances brought by the present work, we note that the argument of the logarithm in the information formula (see Methods) is actually the function that appears in the context theory of classification [23,24] without the probabilities. We here give it an information-theoretic interpretation in terms of marginal and conditional similarity-based entropies. Indeed, in the context-theory of classification, a given item *r* is classified as belonging to a category $c_{1}$ based on the ratio between its similarity to other items in the category $c_{1}$ and its similarity with items in all categories $p\left(c_{1}|r\right)=\frac{\sum_{i}\phi_{r}\left(r_{i}^{1}\right)}{\sum_{j}\sum_{i}\phi_{r}\left(r_{i}^{j}\right)}$. This is similar to the term obtained in the logarithm of the similarity-based information theory, though the latter includes probabilities as well. It thus adds to the context-theory of classification by bringing together in a single formula the contributions of frequency and similarity [6], and by being grounded in the framework of information theory. The advantage of our formulation is that it includes both probabilities and similarity, so the resulting terms can be interpreted as goodnesses.

Third, among the other theoretical advances of this work is the ability to tune the similarity functions with an information-theoretic principle. It could be obvious to some readers that we can model perception with similarity functions. However, we then need an optimization principle that would have a clear interpretation and would not be ad hoc. This is exactly how we proceeded here, by taking without modification an existing theory and applying it directly to the issue of categorization.

Fourth, we show a warping of perceptual space. Similarity measures are wide at the center of categories and narrow their boundaries, akin to the poor within-category and high between-categories discriminability, known as the perceptual magnet effect. We can thus interpret the entropy as the expectation of the goodness surprise. The prototype is the least surprising member of a category. An important feature of our model is that, for a given trade-off α, if categories become larger, then the similarity functions scale likewise. Our model is therefore scale-invariant.

Fifth, as in the experiments mentioned in the introduction, there is an asymmetry between distinguishing two items depending on their order of presentation. When the prototype is presented first, the similarity measure used is wide and the discrimination of a following non-prototype becomes hard. However, if the non-prototype is presented first, the similarity measure is narrow and the discrimination of the prototype is easier. When a first item appears, it sets the comparison level. If it is close to the border of the category, it is much more stringent than when it is in the center.

It has been postulated that the magnitude of the categorical effects depends on the protocol used in the experiments [22]. Here, we suggest that the culprit of these variations may be the trade-off parameter α, which sets the relative importance of discrimination and categorization.

A large body of studies suggests that, in order to account for classification, discrimination, and categorization capacities of human brains, we need to consider them information-processing systems that rely on two essential features: probabilities and similarities. As stated by Quine [41]: “There is nothing more basic to thought and language than our sense of similarity; our sorting of things into kinds”, as also stressed by Tversky and Gati [8]. Only by considering these two factors can we understand the way we perform at various tasks and our cognitive biases. This work attempts at bringing together these three elements: information, probabilities and similarities, thereby mapping Ref. [6]’s paper triptych: “Similarity, Frequency and Category Representation”. It fits in the general attempt to phrase cognition into an information theoretical framework with the addition of a similarity structure.

There have already been attempts to explain categorical perception effects. Lacerda [42] proposed an explanation based on exemplar theory, which only accounts for between-category discrimination though. Following insights from Huttenlocher et al. [43], Feldman et al. [21] proposed a Bayesian model to explain the effect. However, according to the authors, the model only works in the case of unimodal categories and it cannot explain the effect on uniform categories. Our account thus finds its interest by fully explaining the perceptual magnet even in the case of uniform distributions as well as the internal structure of categories in a single framework using both probabilities and similarities.

Note the resemblance with the information-bottleneck method [44]. This approach aims at finding the codewords *X* that maximize the functional:

(12) L=I(X;C)−αI(X;S),

which is interpreted as maximizing the information of *X* about the category *C*, but trying to compress the stimulations *S*. The formulation bears a strong kinship with our method, but, as it is expressed only in Shannon information theoretic terms, it does not take into account the similarities between items. It would thus remain an unconstrained compression and would entail the optimal solution X=C (for low values of α), which could not account for the category structure nor categorical perception.

It is now established that the information bottleneck and RDT are related [38,45]. The trade-off parameters in these two theories are linked to our α. In the field of categorization, this trade-off parameter is present in almost all models, or, more precisely, understanding categorization necessarily requires two opposing phenomena: a faithful encoding of the stimulus and a limiting/grouping phenomenon. For example, in Sims et al. [38], it naturally stems from the application of RDT. Although the link is not completely transparent: (a) minimizing Sims’ cost function is akin to maximizing the similarity-base information, in the sense that it attempts at avoiding confusion or error between two different items; (b) the channel capacity constraint is akin to maximizing the similarity-based equivocation, as it allows elbow room for categories.

One of the strongest points of the work presented here is that our solution is abstract, as we work with similarities between items, without mentioning the type of neural subtrates that could actually implement it. However, the nature of the neurophysiological substrates of the similarity remains an important question to address. Bonnasse-Gahot and Nadal [25] approached the problem with Shannon information theory and, although they focused on partially overlapping categories, our results conceptually matches theirs. In particular, they reached the conclusion that a neural population code optimized for categorization should display a higher resolution of the sensory input near the boundary of categories. This could be done by having more neurons with tuning curves centered near the boundary and/or to have neurons with narrower tuning curves near the boundary. Their actual implementation of a neural code matches our more abstract solution since, more neurons with narrow tuning curves will naturally yield larger neural distances between otherwise similar stimuli. It is interesting to observe that they take the neural code as a fundamental object. An exact quantification of the difference (or lack thereof) between the predictions of the models would require a mapping between population neural activity and item similarities, using, for example, common spike-train or firing rate distances [46].

As for the decision, similarity values can be seen as a level of evidence per unit of time in a drift model of decision-making [47]. This could explain why it takes longer to react in some cases [9,13,48]. This would bring this work closer to the drifting models of decision-making.

### Limitations

One of the main limitations of our work is that we do not explain the origin and birth of categories. We just assumed that categorization is a needed feature of sensory information processing. We take categories for granted and then we try to maximize an objective information theoretical function. Other studies have already attempted to explain the sizes of categories. Komarova et al. [49] proposed an evolutionary mechanism to explain the birth of categories from discrimination. They used a parameter $k_{sim}$ that is very close to our definition of similarity width and that is eventually generalized as a similarity matrix comparable to our family of similarity functions. Regier et al. [32] also attempted to explain categories as an optimal partitioning of a complex irregular perceptual space. It is so far uncertain whether our framework could also bring light on the birth of categories, but it is compatible with the former approaches. We hope in the near future to extend our framework to include the birth of categories. However, it will necessarily involve a measure of the allocation of brain resources to the discrimination of items and thus another trade-off. We believe that the simplicity (one objective function with one trade-off paramater) of the current framework can make it stand alone, notwithstanding these potential extensions.

Another limitation of our work is that it does not include hierarchy of categories, i.e., some items may be part of a subordinate category itself inside a superordinate category (a typical example being “a rabbit is a mammal is an animal”). Corter and Gluck [50] already used information theory to compute what hierarchical level of categorization is optimal to describe an item. Their goal being different from ours, their framework does not include similarities and does not attempt to explain categorical perception. This problem could be dealt with by adding a similarity structure on the set of categories, a feature that can be naturally integrated in the similarity-based information-theoretic framework. However, we are not aware of any experimental studies showing categorical perception or perceptual magnet effects on sub- and super-ordinate categories.

## 5. Conclusions

In this work, we account for both the internal structure of categories and its effects on perception by using a single information-theoretic framework. This framework integrates the notion of pairwise similarity between items into information measures. It provides an optimization principle that, at the same time, maximizes information transmission and compression of the stimulus space. Applying this framework to pre-existing categories to be learned, we derive the notion of goodness of items belonging to a category, categorical perception and the perceptual magnet effect.

The main point of this work is to set forth a single hypothesis about human discrimination in terms of perceived similarity as well as frequencies. There is no assumption on the shape of categories, which can be either bell-shaped or uniform. Nonetheless, we are able to derive the internal structure of categories through a naturally occurring quantity matching goodness, and the main effects of categorical perception with their potential asymmetry, thereby expliciting an ontological link between category structure and categorical perception. In other words, we show that both the goodness and warped perception are consequences of the interaction between pairwise similarities and frequencies, thereby expliciting a previously unknown link between the structure of categories and the perceptual effects of categorization. We show that the discrimination level can be accounted through a parameter α that implements a trade-off between discriminating and confusing pairs of stimulations. Empirical categorical perception is accounted for at low values of this parameter, i.e., when discrimination remains the main objective.

## Figures and Tables

**Figure A1:**
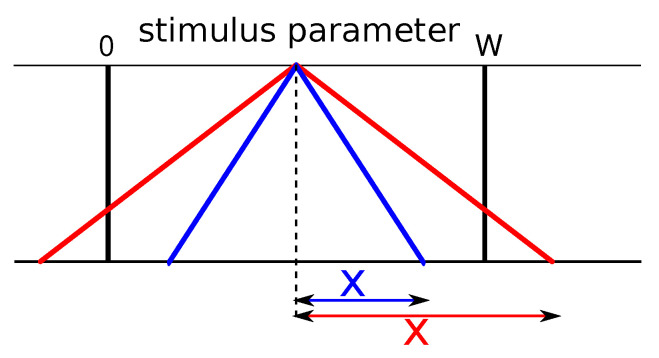
Similarity functions.

**Figure A2:**
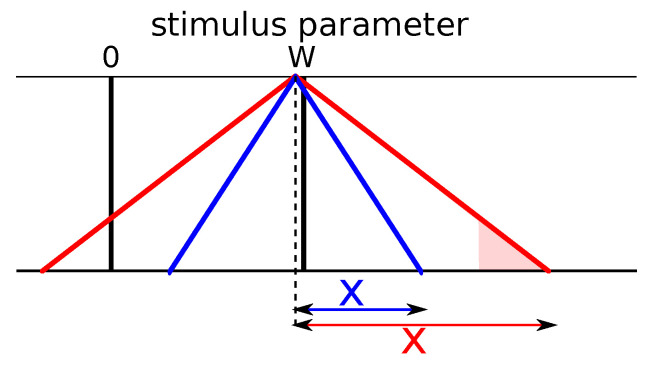
Similarity functions.

## Appendix A

Here, we will give an intuition for the behavior of the similarity width in the case of uniform categories with a triangular kernel (that we use instead of a Gaussian kernel for ease of computation). Let’s look in particular at the optimal similarity width at the center and at the boundary of a category.

### Appendix A.1. Center of Categories

The computations go as follows in the case of *N* uniform categories of width *W* and a triangular kernel of width *x*. Note that p(s|c)=Np(s):

(A1) g(s)=p(s)x.

If x<W/2 (in blue in Figure A1):

(A2) g(s|c)=p(s|c)x,

so we can compute the objective function:

(A3) $$q\left(s;c\right)\propto -\log p\left(s\right)x+\left(1+\frac{\alpha }{\log W}\right)\log p\left(s|c\right)x,$$

which, upon inspection, is an ever-increasing function of *x*. Therefore, the optimal value of *x* must be larger than or equal to $\frac{W}{2}$.

If x>W/2 (in red in Figure A1):

(A4) $$g\left(s|c\right)=p\left(s|c\right)\left(W-\frac{W^{2}}{4x}\right),$$

so we can compute the objective function if x>W/2:

(A5) $$q\left(s;c\right)\propto -\log p\left(s\right)x+\left(1+\frac{\alpha }{\log W}\right)\log p\left(s|c\right)\left(W-\frac{W^{2}}{4x}\right).$$

Deriving this function and equating it to 0 yields:

(A6) $$x_{opt}=\frac{W}{4}\left(2+\frac{\alpha }{\log W}\right),$$

and we find perfect agreement with simulations.

As long as the kernel is positive only within the category, the information remains unaltered, only the conditional entropy is reduced compared to the Kronecker kernel. By increasing the width of the kernel, the conditional entropy is further reduced, but the information is reduced even more. There is thus a trade-off appearing.

If the trade-off parameter α is nil, then the objective is merely to maximize information. In that case, any value of the width smaller than the width of a category is suitable. As soon as α increases and thus minimizing conditional entropy starts to matter, then larger values of the width are optimal.

### Appendix A.2. Boundary of Categories

The argument of the logarithm in the entropy is:

(A7) g(s)=p(s)x,

and in the conditional entropy if x<W (in blue in Figure A2):

(A8) $$g\left(s|c\right)=p\left(s|c\right)\left(\frac{1}{2}+\frac{x}{2}\right).$$

Note that the $\frac{1}{2}$ comes from the fact that the item *s* considered is fully in category *c*.

We can compute the objective function:

(A9) $$q\left(s;c\right)\propto -\log\left[p\left(s\right)x\right]+\left(1+\frac{\alpha }{\log W}\right)\log\left[p\left(s|c\right)\left(\frac{1}{2}+\frac{x}{2}\right)\right].$$

The minimum of this function is at $x=\frac{\log W}{\alpha }$, so that, in the 0<x<W part, there are two local maxima at x=1 and x=W.

If x>A (in red in Figure A2):

(A10) $$g\left(s|c\right)=p\left(s|c\right)W\left(1-\frac{W}{2x}+\frac{1}{2x}\right)$$

so we can compute the objective function:

(A11) $$q\left(s;c\right)\propto -\log\left[p\left(s\right)x\right]+\left(1+\frac{\alpha }{\log W}\right)\log\left[p\left(s|c\right)W\left(1-\frac{W}{2x}+\frac{1}{2x}\right)\right].$$

Deriving this function and equating it to 0 yields:

(A12) $$x_{opt}=\frac{\left(2+\frac{\alpha }{\log W}\right)\left(W-1\right)}{2}.$$

Note that the value of the objective function at $x_{opt}$ is always larger than that at x=W. Thus, it only remains to know when the local maximum at x=1 is larger than that at $x=x_{opt}$.

At x=1:

(A13) $$q1=q\left(s|c\right)\propto -\log p\left(s\right)+\left(1+\frac{\alpha }{\log W}\right)\log p\left(s|c\right).$$

At $x=x_{opt}$:

(A14) $$\begin{array}{cc} q2=q(s|c)\propto & -\log[p\left(s\right)x_{opt}] \end{array}$$

(A15) $$\begin{array}{c} +\left(1+\frac{\alpha }{\log W}\right)\log\left[p\left(s|c\right)W\left(1+\frac{1-W}{2x_{opt}}\right)\right]. \end{array}$$

The difference is:

(A16) $$q2-q1=-\log x_{opt}+\left(1+\frac{\alpha }{\log W}\right)\log\left[W\left(1-\frac{W}{2x_{opt}}+\frac{1}{2x_{opt}}\right)\right].$$

This cancels when:

(A17) $$\alpha =\log W(\frac{\log x_{opt}}{\log[W\left(1+\frac{1-W}{2x_{opt}}\right)]}-1).$$

We solved the last equation numerically and found for W=100 a value of $\alpha_{th}=0.796$.

In the end, the optimal value of *x* depending on α is:

(A18) $$\begin{array}{ccc} x_{opt}= & 1, & \mathrm{if}\;\alpha <\alpha_{th}, \end{array}$$

(A19) $$\begin{array}{ccc} x_{opt}= & \frac{\left(2+\frac{\alpha }{\log W}\right)\left(W-1\right)}{2}, & \;\mathrm{otherwise}. \end{array}$$

With this, we obtain the values of the optimal *x* for all values of α and we observe an exact matching with the numerical results from the core of the article. We see that the optimal value of *x* scales with W−1 (and not with *W*, which creates small departures from scale-independence at very low *W*s).
